# The Lung Vasculature: A Driver or Passenger in Lung Branching Morphogenesis?

**DOI:** 10.3389/fcell.2020.623868

**Published:** 2021-01-14

**Authors:** Yelda Pakize Kina, Ali Khadim, Werner Seeger, Elie El Agha

**Affiliations:** Department of Internal Medicine, Universities of Giessen and Marburg Lung Center (UGMLC), Institute for Lung Health (ILH), Cardio-Pulmonary Institute (CPI), Member of the German Center for Lung Research (DZL), Justus-Liebig University Giessen, Giessen, Germany

**Keywords:** branching morphogenesis, endothelium, VEGF-vascular endothelial growth factor, VEGFR-vascular endothelial growth factor receptor, lung

## Abstract

Multiple cellular, biochemical, and physical factors converge to coordinate organogenesis. During embryonic development, several organs such as the lung, salivary glands, mammary glands, and kidneys undergo rapid, but intricate, iterative branching. This biological process not only determines the overall architecture, size and shape of such organs but is also a pre-requisite for optimal organ function. The lung, in particular, relies on a vast surface area to carry out efficient gas exchange, and it is logical to suggest that airway branching during lung development represents a rate-limiting step in this context. Against this background, the vascular network develops in parallel to the airway tree and reciprocal interaction between these two compartments is critical for their patterning, branching, and co-alignment. In this mini review, we present an overview of the branching process in the developing mouse lung and discuss whether the vasculature plays a leading role in the process of airway epithelial branching.

## Introduction

Cellular rearrangement and pattern formation are integral parts of organogenesis. Cell-cell communication, particularly between the endoderm-derived epithelium and the surrounding mesoderm-derived mesenchyme, sets the stage for lung formation. Such epithelial-mesenchymal interactions are highly defined along the proximal-distal axis in the developing lung. For example, it has been shown that grafting distal lung mesenchyme at the level of the tracheal epithelium leads to ectopic budding and subsequent branching at the grafting site (Alescio and Cassini, 1962). Such bronchial mesoderm, but not non-specific mesoderm, has been shown to be required for branching of the endoderm (Spooner and Wessells, 1970). Additionally, different thresholds of mesenchymal cell abundance have been shown to be required for either epithelial maintenance (in the case of minimal mesenchymal mass), or growth, morphogenesis and differentiation (in the case of larger mesenchymal mass) (Masters, 1976). Later, it was shown that the distal lung mesenchyme is characterized by the expression of fibroblast growth factor 10 (*Fgf10*) (Bellusci et al., 1997) and that *Fgf10*-knockout embryos suffer from lung agenesis as well as other morphogenic and organogenic abnormalities (Min et al., 1998; Sekine et al., 1999; Suzuki et al., 2000; Sakaue et al., 2002; Jaskoll et al., 2005). In agreement with the study mentioned above regarding the role of the mesenchymal mass (Masters, 1976), it was shown that *Fgf10* dosage is critical for the amplification of epithelial progenitors during embryonic lung development (Ramasamy et al., 2007).

**Figure 1:**
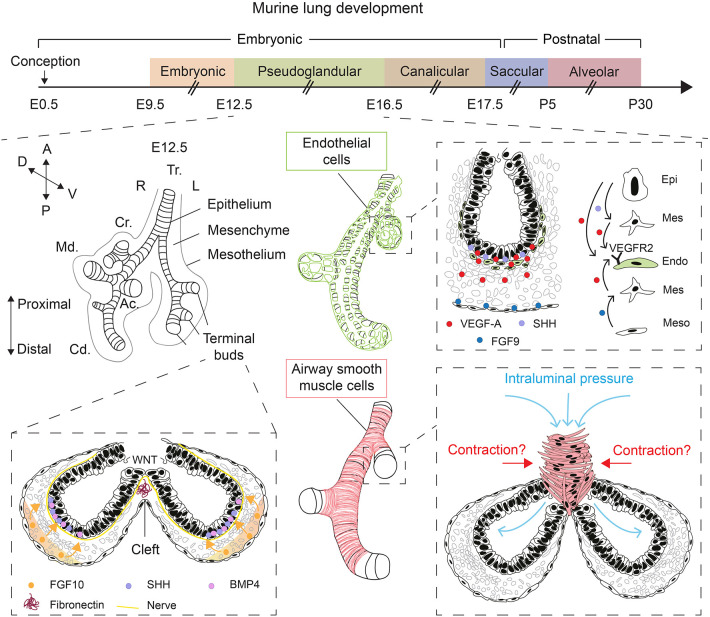
Overview of lung development and various factors controlling epithelial branching morphogenesis. During the pseudoglandular stage of lung development, the lung undergoes successive rounds of branching. FGF10, secreted by submesothelial mesenchymal cells, induces budding and branching of distal terminal epithelial buds. WNT signaling in the distal epithelium induces the deposition of fibronectin at the cleft region, which helps guide bifurcation. Epithelial, mesenchymal, and mesothelial cells cooperate to promote the development of the endothelial network through VEGF-A/VEGFR2 signaling. Intraluminal pressure, innervation, and airway smooth muscle cell differentiation are among many factors that promote branching morphogenesis. Orientation vector: A, Anterior; P, Posterior; D, Dorsal; V, Ventral. Embryonic lung anatomy: Tr, Trachea; R, Right; L, Left; Cr, Cranial lobe; Md, Medial lobe; Cd, Caudal lobe; Ac, Accessory lobe. Cell-type code: Endo, Endothelium; Epi, Epithelium; Mes, Mesenchyme; Meso, Mesothelium. The timeline representation of murine lung development does not correspond to a fixed scale.

Lung development starts with the evagination of the ventral foregut endoderm at embryonic day 9.5 (E9.5) to form the primitive lung buds, thus marking the embryonic stage of lung development that lasts until E12.5 (Figure 1). The lung domain in the foregut is marked by the expression of the transcription factor *Nkx2.1*. Surrounded by the splanchnic mesoderm, the two lung buds start undergoing iterative branching, the hallmark feature of the pseudoglandular stage of lung development (E12.5–E16.5) (Figure 1). Other stages of lung development include the canalicular (E16.5–E17.5), saccular (E17.5 to post-natal day 5; P5), and alveolar stages (P5–P30) (Figure 1). The nomenclature of these stages is mainly based on the histological appearance of these lungs when analyzed by light microscopy (Warburton et al., 2010; El Agha and Bellusci, 2014; Volckaert and De Langhe, 2015).

The mesenchyme is a multifaceted compartment of the developing lung containing a heterogenous mixture of progenitor and differentiated cells, and it represents a reservoir of key growth factors, signaling networks, and even physical forces that direct lung development (Warburton et al., 2005, 2010; Morrisey and Hogan, 2010; El Agha and Bellusci, 2014; McCulley et al., 2015). Mesenchyme topology and dynamics in terms of cellular density and orientation around the growing epithelial buds likely guides the process of morphogenesis, particularly the branching process. Apart from FGF10+ mesenchymal cells that also act as progenitors for differentiated mesenchymal cells such as airway and vascular smooth muscle cells and lipofibroblasts (Mailleux et al., 2005; El Agha et al., 2014, 2017; Al Alam et al., 2015), airway smooth muscle cells (Kim et al., 2015) and nerve cells (Bower et al., 2014; Rhodes et al., 2015) have been proposed to be critical for the branching process, although the involvement of airway smooth muscle cell peristalsis in promoting epithelial branching *in vivo* has been recently questioned (Young et al., 2020) (Figure 1). Wingless-related integration site (WNT) signaling and extracellular matrix (ECM) deposition such as fibronectin—The latter is believed to act as “a rock in the stream” that forces the bifurcation of the growing epithelial tubes (Warburton et al., 2005)—have also been proposed as main drivers of epithelial branching (De Langhe et al., 2005; Kadzik et al., 2014) (Figure 1).

Physical forces have also emerged as an important regulator of epithelial branching (Figure 1). In fact, lung branching has been proposed to be a natural physical consequence of the interaction between cellular layers with surface tension between them (Lubkin and Murray, 1995). Several mathematical models for branching morphogenesis have been proposed such as diffusion-limited growth by FGF (Miura and Shiota, 2002), Turing instability-based model (Menshykau et al., 2012) and mixed-type models (Guo et al., 2014a,b). These models have been reviewed in Miura (2015). Interestingly, it has been shown that increased intraluminal pressure by tracheal occlusion, achieved via cauterization, boosts epithelial branching in air-liquid interface (ALI) cultures of E12.5 embryonic lungs (Unbekandt et al., 2008). Gene expression analysis showed that several key growth factors such as *Fgf10*, sonic hedgehog (*Shh*), and vascular endothelial growth factor a (*Vegfa*) were upregulated whereas sprouty 2 (*Spry2*), a downstream target of FGF10 and an inhibitor of FGF signaling, was downregulated after 48 h of culture (Unbekandt et al., 2008).

During lung development, the vascular tree develops in parallel to its epithelial counterpart where proximal vessels, formed through the process of angiogenesis, and distal vessels, formed through the process of vasculogenesis, fuse to form a continuous vascular lumen around E13/E14 (deMello et al., 1997). The lung vasculature is involved in the regulation of many processes during lung development and its involvement in epithelial branching has been a topic of extensive research. Here, we discuss the various modes of lung branching and whether the lung vasculature guides branching morphogenesis of the lung epithelium during early lung development.

## The Branching Program of the Developing Mouse Lung: Hard-Wired and Stochastic Aspects

Congenital diaphragmatic hernia (CDH) is a defect resulting from impaired development, and therefore incomplete closure, of the diaphragm, leading to the invasion of abdominal organs into the thoracic cavity. CDH newborns suffer from lung hypoplasia and the experimental model of nitrofen-induced CDH in rodents has shown that epithelial branching is indeed impaired in these embryos (Guilbert et al., 2000). This defect therefore favors a model where available space dictates the extent of lung branching. However, the assumption that lung branching morphogenesis is an iterative process that proceeds in a random fashion until the intrathoracic space is filled has already been challenged. Members of various developmental signaling pathways such as FGF, SHH, bone morphogenetic protein (BMP), and WNT signaling have been implicated in this process (Cardoso and Lu, 2006; Warburton et al., 2010) (Figure 1). In a pioneering study, the branch lineage of the bronchial tree and the accompanying branching sequences were analyzed between E11 and E15 using fixed specimens (Metzger et al., 2008). Thorough analysis of such samples showed that the developing lung undergoes three modes of branching defined as domain branching, planar bifurcation, and orthogonal bifurcation. Domain branching sets up the overall shape of individual lobes and is controlled by two patterning systems: A proximal-distal system controlling periodicity/sequence of branching and a circumferential system specifying the position of domains and the order in which they are implemented. Planar bifurcation forms the edges of the lobes and therefore occurs at their tips while orthogonal bifurcation generates the surfaces of the lobes with a 90° rotation in the bifurcation plane between each round of branching. These findings indicate that the branching process is predominantly stereotyped by genetic regulators although there are instances where anomalous branching events, defined as “branching errors” also take place. The branching pattern was therefore proposed to be controlled by a global master routine that controls further subroutines, setting the coupling scheme for each lineage early, and then relaxing and allowing these lineages to continue independently (Metzger et al., 2008).

The paradigm that lung branching morphogenesis is stereotyped and hard-wired was challenged by Blanc et al. (2012) who carried out three-dimensional (3D) reconstruction of whole right cranial lobes between E11.25 and E13.5. The authors concluded that beyond early branching generations, the branching stereotypy loosens up, and epithelial buds simply try to homogenously fill the surrounding mesenchymal space. The dynamic changes of mesodermal shape seemed to influence both the branching pattern and branching rate, indicating that branching of the endoderm is basically connected to local changes of mesoderm growth (Blanc et al., 2012). Those findings indicated that the branching mode of the epithelial tree is unlikely to be specified and predefined by a rigid, global genetic program.

## VEGF-A/VEGFR2 Signaling Positively Regulates Epithelial Branching Morphogenesis

VEGFs are critical ligands for the development of blood vessels from hemangioblasts, a population of hematopoietic, and endothelial progenitor cells. These ligands are produced by both epithelial and mesenchymal cells at E12.5. Using organotypic cultures of E11.5 lung explants grown in an ALI for up to 4 days, it was shown that VEGF-A is deposited in the subepithelial mesenchyme adjacent to the branching distal epithelial buds where it upregulates endothelial cell markers and promotes vasculogenesis (Healy et al., 2000) (Figure 1). Such expression pattern hinted at a possible role for VEGF-A in coordinating airway branching and blood vessel formation during early lung development. Later work demonstrated that epithelium-derived SHH and mesothelium-derived FGF9 cooperate to induce *Vegfa* expression in both the subepithelial and submesothelial mesenchyme, that in turn acts on vascular endothelial growth factor receptor 2-positive (VEGFR2+; aka fetal liver kinase 1-positive or FLK1+) endothelial progenitors to induce vasculogenesis, thus contributing to the formation of the capillary plexus (White et al., 2007) (Figure 1). Interestingly, overexpression of *Vegfa* in distal epithelial cells, but not in proximal airways, disrupts the peripheral vascular network, and impairs epithelial branching (Akeson et al., 2003). The latter findings highlight the importance of the crosstalk between the epithelium and endothelium in the distal lung and that disturbance of the spatial and temporal control of VEGF-A signaling has implications for branching morphogenesis.

Since lung development proceeds in a hypoxic environment inside the uterus, it was hypothesized that low oxygen levels represent an important factor for lung branching morphogenesis as well as vascular development. Indeed, E11.5 embryonic lung explants grown in ALI at 3% oxygen exhibited enhanced numbers of terminal epithelial branches compared with controls cultured at 20% oxygen (van Tuyl et al., 2005). Interestingly, the spatial expression patterns of mesenchymal *Fgf10* and epithelial *Bmp4* did not seem to be affected by hypoxia. Moreover, vascular development was boosted by hypoxia as evident by the extensive endothelial network that extended from the trachea to distal epithelial tips. Knock down of *Hif1a* and *Vegfa* led to dramatic impairment of vascularization in these lung explants while exogenous VEGF treatment rescued the phenotype in *Vegfa–*, but not *Hif1a–*, knockdown explants. Importantly, arrested vascular development coincided with significant simplification of the epithelial tree indicating that the disturbance of vascular network assembly disrupts epithelial branching (van Tuyl et al., 2005).

The positive effect of VEGF signaling on epithelial branching has been shown to be mediated by endothelial-epithelial crosstalk (Gebb and Shannon, 2000; Del Moral et al., 2006). Using organotypic cultures of E11.5 lung explants, it was shown that treatment with VEGF enhanced the number of terminal epithelial branches in parallel to increased epithelial and mesenchymal proliferation (Del Moral et al., 2006). VEGF did not have a direct effect on isolated endoderm. The impact on lung branching coincided with the upregulation of *Flk1, Sftpc*, and *Bmp4* and the downregulation of *Spry2/4*, which are known inhibitors of FGF signaling. Conversely, knock down of *Flk1* led to reduced terminal branching with downregulation of *Flk1, Sftpc*, and *Bmp4* in parallel to upregulation of *Spry2*/4. Loss of *Flk1* expression also led to significant reduction of proliferation in both the epithelium and mesenchyme (Del Moral et al., 2006). Those findings suggested that the vasculature is an important factor for promoting epithelial branching during early lung development.

In another study, the effect of *in vivo* vascular depletion on the branching stereotypy and the coordination between the branching of epithelial and vascular tubes leading to their co-alignment was studied (Lazarus et al., 2011). A loss-of-function approach involving the overexpression of a dominant-negative, decoy vascular endothelial growth factor receptor 1 (VEGFR1) to inhibit the effect of VEGF signaling between E6.5 and E12.5 was employed. E12.5 lungs showed that vascular ablation caused fewer, more dilated airway branches in addition to ectopic branching events compared with control lungs (Lazarus et al., 2011). This was accompanied by upregulation of *Spry2* and downregulation of *Shh*, the gene encoding its receptor (patched 1 or *Ptc1*) and downstream target glioma-associated oncogene 1 (*Gli1*). Importantly, domain branches, as described by Metzger et al. (2008), requiring a change in the branching plane were preferentially affected by vascular depletion. These branching abnormalities coincided with impaired expression of the branching mediator *Fgf10* and branching regulators *Shh* and *Spry2* (Lazarus et al., 2011). Additionally, treatment of E11.5 ALI cultures with a VEGFR2 intracellular inhibitor led to vascular depletion coupled to a 30% reduction in planar bifurcations and a 70% reduction in orthogonal bifurcations (Lazarus et al., 2011). Remarkably, the *in vivo* experiments showed that airway branching and the 3D architecture of the lung seemed to be fully rescued as a consequence of revascularization due to termination of VEGF blockade between E12.5 and post-natal day 14 (P14), indicating that the branching arrest was reversible and that vascular regain was at least permissive for kick-starting epithelial branching morphogenesis (Lazarus et al., 2011).

Using organoid cultures, it was shown that human umbilical vein endothelial cells (HUVECs) facilitated the generation of branched bronchioalveolar organoids from human bronchial epithelial cells (Franzdóttir et al., 2010). Further analysis confirmed that airway epithelial cells were polarized, and that inhibition of FGF signaling via pharmacological intervention inhibited the branching process (Franzdóttir et al., 2010).

Another culture system involves the engraftment of E12.5 lungs under the kidney capsule of adult host mice. Using this model, it was shown that inhibiting VEGF-A activity by injecting host animals with a dominant-negative decoy mFLT(1–3)Ig led to the inhibition of both vascular development and epithelial branching (Zhao et al., 2005).

Collectively, there is strong evidence suggesting that the vascular network is critical for proper epithelial morphogenesis, at least in part by affecting the spatial expression pattern of key genes involved in the branching process. In the next section, evidence suggesting that the lung epithelium is capable of undergoing branching morphogenesis in the absence of the vascular system will be presented.

## The Lung Epithelium is Capable of Branching in the Absence of Endothelial Cells

Despite the general notion that the lung vasculature has a strong influence on epithelial branching in the developing lung, several lines of evidence suggest that the vasculature is dispensable for epithelial branching. For example, it has been reported that epithelial branching proceeds even in the absence of endothelial cells *in vitro* (Havrilak and Shannon, 2015). The authors used ALI cultures of E12.5 lung explants to show that treatment with various VEGFR inhibitors did not impair the branching process after 48 h of *ex vivo* culture. Loss of endothelial cells as a result of such treatment did not seem to impair epithelial, smooth muscle or pericyte differentiation. Additionally, the authors elegantly showed that distal mesenchymal cell suspensions that had been depleted of endothelial cells were capable of promoting the formation of branched structures when combined with distal epithelial cell suspensions and allowed to grow on a membrane for 5–7 days in the presence of SHH stimulation (Havrilak and Shannon, 2015). Moreover, they also showed that primary lung endothelial cells isolated from E12.5 lungs or human lung microvascular endothelial cells (HMVEC-L) were not capable of promoting branching of epithelial rudiments when cultured in basement membrane extract as opposed to the positive control cultured in the appropriate medium (Havrilak and Shannon, 2015). Those findings argue that the endothelial component is dispensable for epithelial morphogenesis and branching. Follow-up work showed that pharmacological inhibition of VEGFR at E8.5 did not affect early lung specification and bud formation (Havrilak et al., 2017). On the other hand, analysis of *Flk1*-knockout embryos that do not develop endothelial cells revealed impaired pulmonary specification in the mutant endoderm likely due to delayed development as such mutants were able to catch up with the controls in terms of respiratory specification and bud formation when cultured *ex vivo* (Havrilak et al., 2017).

Another line of evidence for the dispensability of endothelial cells for lung branching comes from the organoid field. Human pluripotent stem cells (hPSCs) can be used to generate endoderm spheroids, and further conditioned to give rise to branched lung organoids in the absence of endothelial cells (Miller et al., 2019). Even earlier studies using isolated endoderm have shown that recombinant growth factors such as FGF1 or FGF10 are sufficient to induce epithelial branching (Nogawa and Ito, 1995; Bellusci et al., 1997). Co-culturing bronchioalveolar stem cells (BASCs) characterized by dual expression of airway and alveolar markers (*Scgb1a1* and *Sftpc*, respectively) with appropriate resident mesenchymal cells (CD45- CD31- EpCAM- SCA-1+) leads to the formation of highly branched bronchioalveolar organoids that are reminiscent of the branching lung (Vazquez-Armendariz et al., 2020). Last but not least, several theoretical works have shown that FGFs *per se* drive lung branching (Miura and Shiota, 2002; Clément et al., 2012). These data, as well as others, strongly suggest that the epithelium is highly responsive to mesenchymal signals that instruct branching morphogenesis and that the vasculature does not seem to play a leading role nor is absolutely required for epithelial branching.

## Discussion

Tissue interactions represent a hallmark feature of lung organogenesis, and various cellular, molecular, biochemical, and physical mechanisms have been implicated in this developmental process. In this context, the interplay between epithelial, mesenchymal, mesothelial, and endothelial cells is absolutely critical for coordinating the development of the various constituent tissues within the lung. As highlighted above, vascular ablation *in vivo* mainly disrupts vascular development but also perturbs epithelial branching patterns. The observation that endothelial cells appear to be dispensable for epithelial rearrangement and branching suggests that the vascular system seems to play a modulatory role and is mainly involved in downstream patterning of airway branching during the course of lung development. The influence of the lung endothelium might be mediated by endothelium-derived factors that modulate ECM properties such as stiffness or heparan sulfate proteoglycan composition. Heparan sulfates are known to interact with diffusible proteins such as FGF10 and create growth factor gradients that instruct epithelial patterning (Izvolsky et al., 2003; Warburton et al., 2010). Therefore, it is plausible that the endothelium creates a permissive or modulatory environment that impacts branching patterns along the growing epithelial tubes. Given the close association between the distal epithelium and the endothelial layer, it is possible that interaction between the two cellular domains across the basement membrane is mediated by paracrine-acting growth factors (other than VEGF-A/VEGFR2 signaling) and downstream (positive and negative) feedback-loop mechanisms. Epithelial-endothelial interaction might also be mediated by ECM-integrin signaling or even physical forces arising from sprouting of endothelial cells leading to the engulfment of terminal buds by the capillary plexus.

Despite the widespread use of the elegant ALI organotypic culture system, one of its limitations is that it heavily distorts the 3D structure of embryonic lungs and leads to severe flattening on polycarbonate membranes. This issue might mask potential deleterious effects for vascular ablation on the 3D patterning of epithelial branches. Moreover, oxygen concentration, the absence of the systemic blood circulation and extrapulmonary cells such as bone marrow-derived cells and loss of nerve connections collectively alter the physiological, biochemical and biophysical context, and certainly do not mimic the *in-utero* environment. On the other hand, the majority of the *in vivo* genetic approaches that have so far been employed do not perturb the endothelium in a cell-autonomous fashion, which opens the door for potential non-specific effects on non-endothelial cells. Future studies designed to selectively manipulate/ablate endothelial cells in the lung during defined intervals with minimal off-target effects *in vivo* might unveil novel mechanisms related to epithelial-endothelial crosstalk along the proximal-distal axis and their impact on lung branching.

## Author Contributions

YK, AK, WS, and EEA drafted and edited the manuscript. All authors contributed to the article and approved the submitted version.

## Conflict of Interest

The authors declare that the research was conducted in the absence of any commercial or financial relationships that could be construed as a potential conflict of interest.
